# Estrogen receptors involvement in intervertebral discogenic pain of the elderly women: colocalization and correlation with the expression of Substance P in nucleus pulposus

**DOI:** 10.18632/oncotarget.15421

**Published:** 2017-02-16

**Authors:** Xiao-Xing Song, Sheng Shi, Zhen Guo, Xin-Feng Li, Bu-Wei Yu

**Affiliations:** ^1^ Department of Anesthesiology, Rui Jin Hospital, School of Medicine, Shanghai Jiao Tong University, Shanghai, China; ^2^ Department of Orthopaedic Surgery, Ren Ji Hospital, School of Medicine, Shanghai Jiao Tong University, Shanghai, China; ^3^ Department of Orthopaedic Surgery, Yang Pu Hospital, Tongji University, Shanghai, China

**Keywords:** intervertebral disc, pain, estrogen receptor, nucleus pulposus, substance P, Gerotarget

## Abstract

Estrogenic modulation of pain is an exceedingly complex phenomenon. However, whether estrogen is involved in discogenic low back pain still remains unclear. Here, immunoreactivity staining technique was used to examine the expression level of the estrogen receptors (ERα and ERβ) and a pain related neuropeptide, Substance P in the lumbar intervertebral discs to analyze the relationship between the ERs and Substance P. Nucleus pulposus tissues of 23 elderly female patients were harvested during spinal surgeries and made to detect the immunoreactivity staining of ERα, ERβ and Substance P. The colocalization and intensities of ERs and Substance P were explored and evaluated respectively. The correlations between changes of ERα, ERβ and Substance P were also assessed.Our results revealed that Substance P colocalized with ERα and ERβ both in cytoplasm and nucleus of the nucleus pulposus cells. HSCORE analysis indicated that Substance P negatively correlated with both ERα and ERβ expression. Collectively, the crosstalk between ERs and Substance P might exist in the disc tissue. Estrogen-dependent pain mechanism might partly be mediated through ERs and Substance P in the nucleus pulposus of the elderly females. Estrogen and its receptors might be drug targets in discogenic low back pain diseases.

## INTRODUCTION

Recently, estrogen was indicated to play an important role in pain modulation [1]. Low back pain was commonly reported after menopause and suggested to be linked to estrogen in women. However, observational studies examining exogenous estrogen's influence on back symptoms provided mixed results. Some clinical studies examining relationships between exogenous estrogen use and back pain symptoms reported a negative effect [2–4], whereas, a favorable effect [5] and no clear association [6] has also been indicated. The mechanisms by which estrogen modulates back pain appear to be highly complex.

The intervertebral disc (IVD) related pain is a significant proportion in cases of chronic back pain [7], imparting a large socioeconomic burden on the healthcare system. However, the mechanism of discogenic low back pain remains unclear [8, 9]. Some investigations showed the involvement of estrogen on IVD health [10]. Besides, the estrogen decrease during menopause contributes to the progressive decrease of the IVD height [11]. The estrogen receptors (ERs), ERα and ERβ, are the main mediators of estrogen action. ERβ gene expression has been detected in human IVD annulus cells [12], but the expression of ERα and the relationship between estrogen receptors and discogenic pain have not been established.

Substance P, secreted by nerves and inflammatory cells, is a tachykinin that serves as a neurotransmitter and a sensory marker related to pain [13], and might be involved in the inflammation pain of several tissues [14–16]. Substance P have been also described within the lumbar IVD in humans. Hence, IVD might be a pain generator [17]. Meanwhile, the influence of estrogen on the Substance P expressions in the synovium of osteoarthritis joints has been reported, which provided valuable insights into the involvement of estrogen in chronic osteoarthritis pain [18]. In this study, the relationship between the expressions of estrogen receptor and Substance P were investigated in IVD tissue.

## RESULTS

### ERα, ERβ and Substance P immunoreactivity in human nucleus pulposus

**Figure 1:**
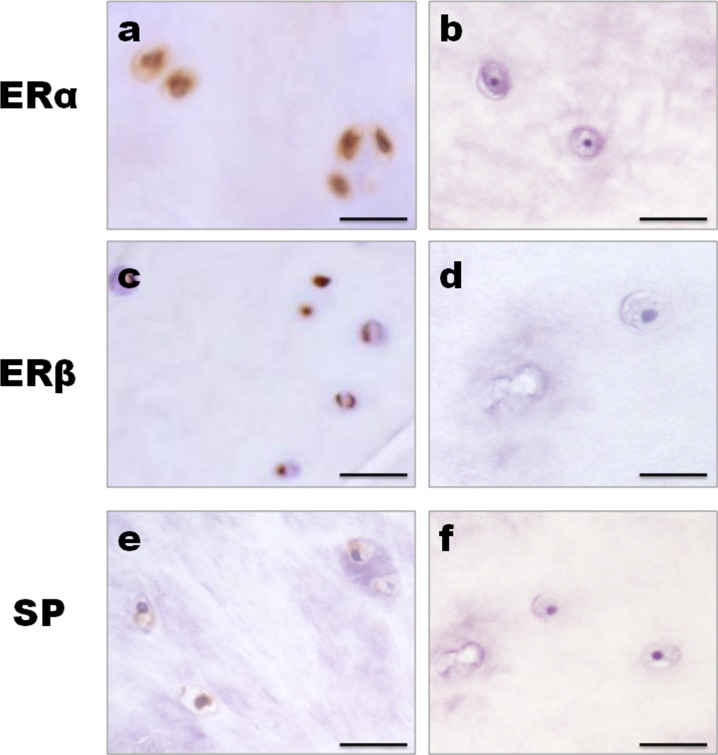
Control studies for immunohistochemistry The IVD tissues of the elderly men were used as the control. Positive immunoreactivity produced a brown coloration. **a**., **c**., **e**. Both cytoplasmic and nuclear staining of ERα, ERβ and SP immunoreactivity were observed in the nucleus pulposus cells. **b**., **d**., **f**. No staining could be observed when the antibody was pre-incubated with a blocking peptide. Bar represents 50 μm; SP, Substance P.

**Figure 2:**
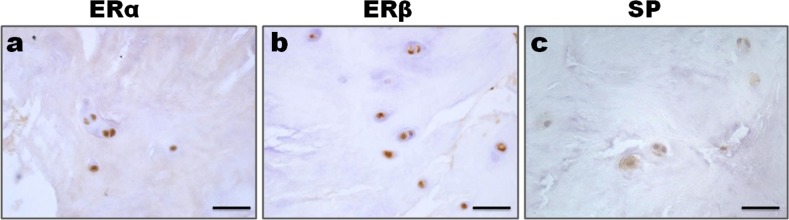
Expression of ER and SP in the nucleus pulposus cells **a**., **b**. Both cytoplasmic and nuclear staining of ERα and ERβ immunoreactivity were observed in the nucleus pulposus cells. **c**. SP immunoreactivity presented with a cytoplasmic and nuclear staining. Bar represents 50 μm; SP, Substance P.

Immunopositive staining was observed for ERα, ERβ and Substance P protein. Cellular staining for ER and Substance P was identified in the native chondrocyte-like cells of the nucleus pulposus tissue in IVD. Control immunohistochemistry study revealed that a brown coloration as a positive immunoreactivity of ERα, ERβ and Substance P in the nucleus pulposus cells of the elderly men. No staining was detected when antibody was pre-incubated with a blocking peptide (Figure 1). As shown in Figure 2, both cytoplasmic and nuclear staining of ERα, ERβ and Substance P immunoreactivity were observed in the nucleus pulposus cells of the female.

### Colocalization of Substance P with ERα or ERβ in human nucleus pulposus

**Figure 3:**
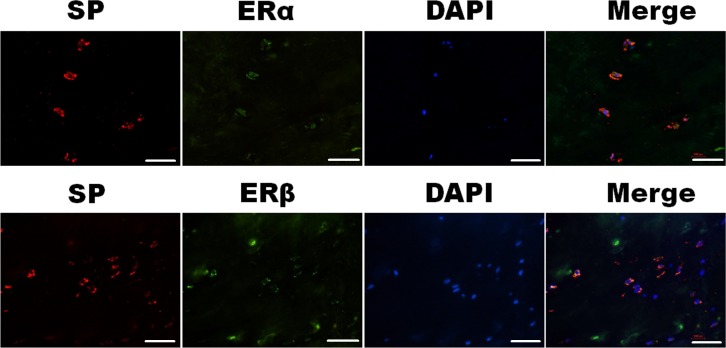
Simultaneous expression of SP (red) and ERα (green) or ERβ (green) in the nucleus pulposus cells were detected under confocal microscopy Photomicrographs showed SP colocalized with ERα and ERβ at the cytoplasm and nuclear in the nucleus pulposus cells. Bar represents 50 μm; SP, Substance P.

Dual-label confocal immunofluorescence examination was used to investigate the simultaneous expression of Substance P with ERα and ERβ in the nucleus pulposus. Colocalizations of Substance P (red) and ERα (green) or ERβ (green) were demonstrated in Figure 3. Substance P colocalized with ERα and ERβ at both cytoplasm and nucleus in the nucleus pulposus cells.

### Correlation analysis between changes of ERs and Substance P in the nucleus pulposus

**Figure 4:**
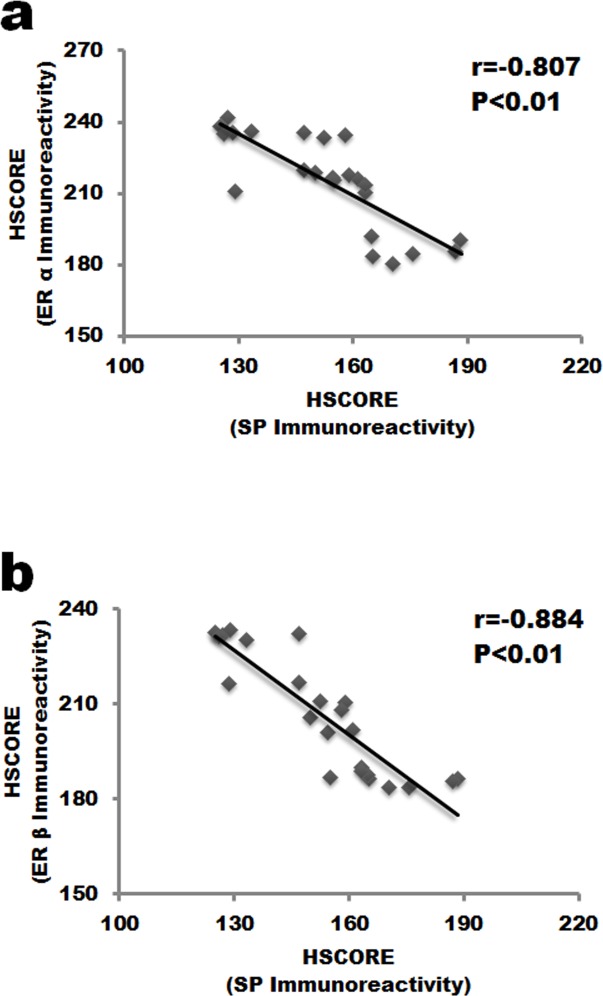
Correlation analysis between the expression of ERs and SP in the nucleus pulposus cells **a**. ERα staining negatively correlated with SP expression in IVD tissue. **b**. SP staining was negatively associated with ERβ. SP, Substance P.

Coexpression of ERs and Substance P in human nucleus pulposus indicated that there might exit correlation between them. To determine the potential relationship among them, correlation analysis was performed (Figure 4). HSCORE value of Substance P staining negatively correlated with both ERα (r = -0.807, *P* < 0.01, Figure 3a) and ERβ (r = -0.884, *P* < 0.01, Figure 3b) HSCORE data.

## DISCUSSION

It has been reported that estrogen could influence the metabolism of the IVD and associated structures such as bone and articular cartilage [19, 20]. Much more prevalence of chronic pain conditions in women than men suggested a possible link between estrogen and pain pathogenesis [21]. However, the role of estrogen in regulating nociception remains unclear. Thus, the present study aimed to evaluate whether a possible correlation exit between ERs and Substance P in human IVD tissue. The data indicated that Substance P colocalized with both ERα and ERβ in the nucleus pulposus of the elderly females. HSCORE analysis showed that the change of Substance P protein staining was negatively associated with that of ERα and ERβ protein. Estrogen might directly regulate pain in IVD through ERs. ERs were partly involved in intervertebral discogenic pain in the elderly women.

Some literature showed that menopause can accelerate IVD aging. Estrogen can prevent the degeneration and preserve the health of the IVD. Estrogen deficiency might induce disc degeneration in post-menopausal women and produce low back pain associated with disc degeneration [22]. The presentation of ERα and ERβ in nucleus pulposus in our study confirmed that effects of estrogen on IVD metabolism could be direct. The influence of estrogen might be cell- and site specific [23, 24]. 17β-estradiol increased the binding of muscimol to GABA-A-receptors in the female rat spinal cord and decreased the contents of Substance P [25]. Estradiol could exert antinociceptive effects *via* an interaction with α-2 receptors and serotonin receptors in a rat model of inflammatory hyperalgesia [26]. In studies on either ERα or ERβ knockout mice, the sex difference in basal mechanical pain threshold and inflammatory hypersensitivity was eliminated suggesting a fundamental role of ERα and ERβ in nociception in female mice [27]. Both ERα and ERβ appeared to be involved in pain transmission and modulation but might be acting at distinct levels of the pain pathways [28]. However, the substantial contribution of estrogen to the IVD related pain remains unclear, although hormonal factors are generally associated with chronic musculoskeletal pain in women [29–34]. Osteoarthritis (OA) is also more common in women after menopause. As for female IVD, females might be more susceptible to disc degeneration than males. MR-based disc degeneration grading study of the elderly subjects demonstrated that females had more severe disc degeneration than males at all lumbar levels [35]. Clinical studies also noted that women seem to be more susceptible to the deterioration of spine [36]. A randomized, placebo-controlled, clinical trial reported that estrogen-alone use in postmenopausal women could reduce the frequency of joint pain [21]. In women with breast cancer, the arthralgia related to aromatase inhibitor therapy has been noted [37]. Our study demonstrated that Substance P was negatively associated with that of ERα and ERβ protein in IVD. Estrogen-dependent pain mechanism might be mediated through ERs in the nucleus pulposus of females. A novel estrogen-dependent mechanism should be studied further in chronic musculoskeletal pain, including pain generated from IVD.

Colocalization of Substance P with both ERα and ERβ in the nucleus pulposus of females were demonstrated in our study. There might be crosstalk between Substance P and ERs. It has been reported that Substance P expression increased in the painful degenerate IVD [38]. Some study also reported that estradiol could mediate sex differences in formalin-evoked substance P release in rats [39]. In the synovium of OA joints, estrogen might partly influences intraarticular neurogenic inflammation by modulating the expressions of Substance P [18]. 17β-estradiol could decrease the contents of Substance P in female rats [25]. Therefore, we speculated that estrogen might influence the expression and/or the release of Substance P through ERs in IVD. Substance P can trigger neurogenic inflammation, including vasodialtion, plasma extravasation and mast cell degranulation, thereby leading to hyperalgia [40]. The peripheral blood level of Substance P increased in women with burns [41], whiplash injury [42], femoral neck fracture [43], and complex regional pain syndrome [44]. In the synovium of OA joints, estrogen might partly influence intraarticular neurogenic inflammation by modulating the expressions of Substance P [18]. Ovarectomy could induce hyperalgesia, and treating with estrogen could counter-balance the lost of ovaries [45–47]. The present study also support an anti-nociceptive role for estrogen in pain sensitivity in IVD tissue. It has been demonstrated that exogenous estrogen therapy attenuates the hyperalgesic state in ovariectomized animals, but its mechanism should be fully clarified, especially in IVD tissue.

Several limitations should be noted in the present study. First, healthy disc tissue was not included in this study. The expression features of ERα, ERβ and Substance P might be different between healthy and pathologic status. Second, Semiquantitative assessment of immunohistochemical results is highly subjective. Although both investigators used the same grading in our study, there also has a potential for subjective misinterpretation. Third, there was lack of pain assessment related to the findings in the present study. Therefore, future studies using a larger sample size on the ERα, ERβ and Substance P expression related to pain assessment should provide more meaningful data.

In summary, ERα, ERβ and Substance P are expressed in human IVD tissue of the elderly woman. Substance P colocalized with both ERα and ERβ in the nucleus pulposus of woman. Substance P expression level negatively correlated with that of both ERα and ERβ. Estrogen might directly regulate pain in IVD through ERs. Estrogen-dependent pain mechanism might partly be mediated through the crosstalk between ERs and Substance P in the nucleus pulposus of the elderly females.

## MATERIALS AND METHODS

### Patients and samples harvest

Human IVD tissue was obtained at surgery, with informed consent of the patient or relatives. Study protocols were approved by the Institutional Review Board and the local Ethics Committee of our institution. Each subject was identified only by number. Twenty-three elderly female patients(mean age: 69.3± 2.6 years; ranging 65-77 years), who underwent disc excision and/or posterior lumbar interbody fusion for lumbar disc degeneration and discogenic low back pain were selected. All women were in post-menopause status. Discography was used for diagnosis. Patients with tumor, infection, immunological and endocrine disease were excluded. All samples were obtained from L4/5 or L5/S1 levels. No multiple disc levels were used in the present study. After removing the herniated disc tissue, nucleus pulposus tissues at the center of an intervertebral disc were harvested during spinal surgeries as previously reported [48, 49]. Cartilaginous endplates and annulus fibrosus tissues were discarded to ensure the identity of the nucleus pulposus tissue as much as possible [50–55]. The nucleus pulposus tissue was immediately washed with phosphate-buffered saline (PBS) (pH 7.4) and was routinely fixed in 4% paraformaldehyde followed by embedded in paraffin for immunostaining.

### Immunohistochemistry

Immunohistochemistry was performed according to our previously described procedure [56, 57]. Paraffin sections (5μm thick) of the IVD tissue were treated with xylene to remove paraffin and rehydrated in graded alcohol baths followed by three rinses with PBS. Slides were immunostained with the streptavidin-biotin peroxidase (SABC) technique. The IVD tissues of the elderly men were used as the control for ERα, ERβ and Substance P expression. Control experiments were incubated with the antibody pre-incubated with a blocking peptide.

Antigen retrieval was performed by heating the sections in 10 mmol/L citrate buffer (pH 6.0) up to 95°C for 10 min and allowing them to cool down to room temperature for 20 min. Then sections were incubated in 1% H2O2 for 15 min for blocking the endogenous peroxidase activity. After preincubation with 5% normal goat serum (Vector, S-1000) for 30 min at room temperature, sections were incubated overnight at 4°C at room temperature with rabbit polyclonal antibody of ERα (Santa Cruz, sc-543, 1:100 dilution), ERβ (Abcam, ab3576, 1:100 dilution) and Substance P (Boster, BA0126, 1:100 dilution). Then sections were incubated with the corresponding biotinylated goat anti-rabbit IgG (Vector, BA-1000), applied for 30 min at a dilution of 1:200, followed by a triple wash in PBS. Finally, the sections were incubated in ABC complex (Vectastain ABC kit, Vector Cat#PK-6100) for 30 min at room temperature. Staining was visualized with DAB peroxides substrate solution for 3 min, followed by rinsing in distilled water briefly. The slides were dehydrated in graded ethanol, cleared in xylene, and mounted with Permount medium after counterstaining with Gill's hematoxylin solution for 3 min.

### Dual label immunofluorescence staining

Colocalization of ERs and Substance P was explored using immunofluorescence study. As we previously described [58], the slides were immersed to a boil (99°C~100°C) in 0.01M sodium citrate buffer (pH 6.0) for 10 minutes. After the nonspecific binding was blocked with normal 5% goat serum (Vector, S-1000), slides were incubated overnight with a rabbit anti-Substance P antibody (Boster, BA0126, 1:100 dilution), followed by the Alexa Fluor^®^ 594 Goat anti-rabbit (Invitrogen, A11037) incubation 30 min at room temperature in dark, washed with PBS 3*3 times. Then added rabbit anti-ERα antibody (Santa Cruz, sc-543, 1:100 dilution) incubating 30min at room temperature in dark, followed by Alexa Fluor^®^ 488 Goat Anti-Rabbit IgG (Invitrogen, Cat:A11034) fluorescent conjugated secondary antibodies for 30min, washed with PBS 3*3min times. Finally, the slides were mounted with 2-(4-amidinophenyl)-6-indolecarbamidine dihydrochloride (DAPI) mounting solution (5 μg/ml) (Invitrogen, P36935). As for the rabbit anti-ERβ antibody (Abcam Inc., ab3576, 1:100 dilution), incubated overnight at 4°C. On the following day, the slides were washed with PBS and then incubated with Alexa Fluor^®^ 488 Goat Anti-Rabbit IgG 30min at room temperature, then washed with PBS and followed by DAPI staining as the same before. All sildes were evaluated by 50i Nikon microscope in dark. The nucleus were visualized blue using a filter 330-380 nm and positive labeled expression with green by filter 465-495 nm, with red by filter 530-600 nm. Control staining was performed on adjacent serial sections and consisted in replacing the primary antibody with 0.1% BSA in PBS.

### Semi-quantitative analysis of ERs and Substance P staining intensities

The intensities of ERα, ERβ and Substance P immunoreactivity were semi-quantitatively evaluated using the following intensity categories: 0, no staining; 1, weak but detectable staining; 2, moderate or distinct staining; 3, intense staining. We normalized images background and variability in staining based on the negative control of the samples. For every sample, after summing the percentages of cells that stained at each intensity group and multiplying that by the weighted intensity of the staining, a HSCORE value was derived. The formula HSCORE = Σii×Pi, where i represents the intensity scores, and Pi is the corresponding percentage of the cells, was used for calculation. Three tissue sections from each IVD sample were randomly obtained and five randomly selected areas were evaluated for every tissue slide under the microscope with 200×original magnification. The percentage of the cells at each intensity group within these areas was determined by two investigators blinded to the type of the IVD tissues. The average score was used.

### Statistical analysis

Data from ERα, ERβ and Substance P immunohistochemistry scores in the IVD tissue were normally distributed as tested by Kruskal-Wallis (H) test. Quantitative data regarding HSCORE analysis are presented as mean ± SD. The correlations between changes of ERα, ERβ, ERα/ERβ and Substance P were assessed by means of Pearson's correlation coefficient test. A *P* value of < 0.05 was considered statistically significant. Statistical analysis was performed using the SPSS 17.0 statistical package (SPSS Inc., USA).
